# Supplementary material for: Dynamic Europa ocean shows transient Taylor columns and convection driven by ice melting and salinity
Source: Nat Commun. 2021 Nov 4;12:6376. doi: 10.1038/s41467-021-26710-0 (PMC8569204; doi:10.1038/s41467-021-26710-0)
Supplement: Supplementary file 1 — Supplementary Information file [file 41467_2021_26710_MOESM1_ESM.pdf]

Supplementary Information:
Dynamic Europa ocean shows transient Taylor columns and convection driven by ice melting and salinity

Yosef Ashkenazy[1] & Eli Tziperman[2]

[1]*Department of Solar Energy and Environmental Physics, The Blaustein Institutes for Desert Research, Ben-Gurion University of the Negev, Midreshet Ben-Gurion, 84990, Israel.*

[2]*Department of Earth and Planetary Sciences and School of Engineering and Applied Sciences, Harvard University, 20 Oxford Street, Cambridge, Massachusetts 02138, USA.*

Supplementary Figures

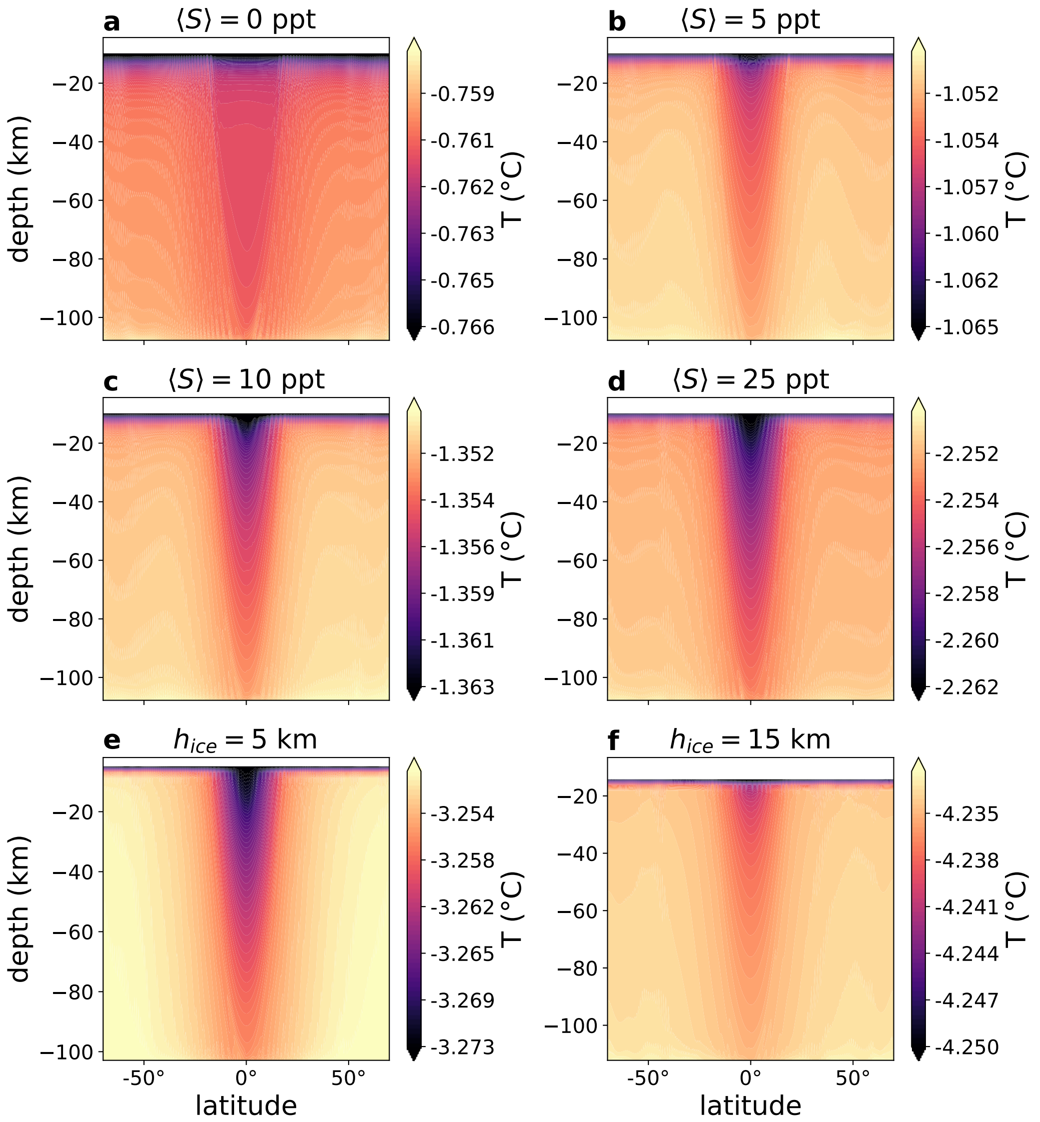

Supplementary Figure 1: **Sensitivity tests–temperature.** 2d latitude-depth snapshots of temperature, T, for mean salinity of **a** 10^{-6} ppt, **b** 5 ppt, **c** 10 ppt, **d** 25 ppt, and ice thickness of **e** 5 km (corresponding to ocean bottom heat flux of 100 mW m^2), and **f** 15 km (corresponding to ocean bottom heat flux of 33 mW m^2). Note that in panel a, with the vanishing mean salinity, the source of the bottom dense water is the bottom heating combined with the water anomaly at this range of temperatures, which leads to a density increase with heating.

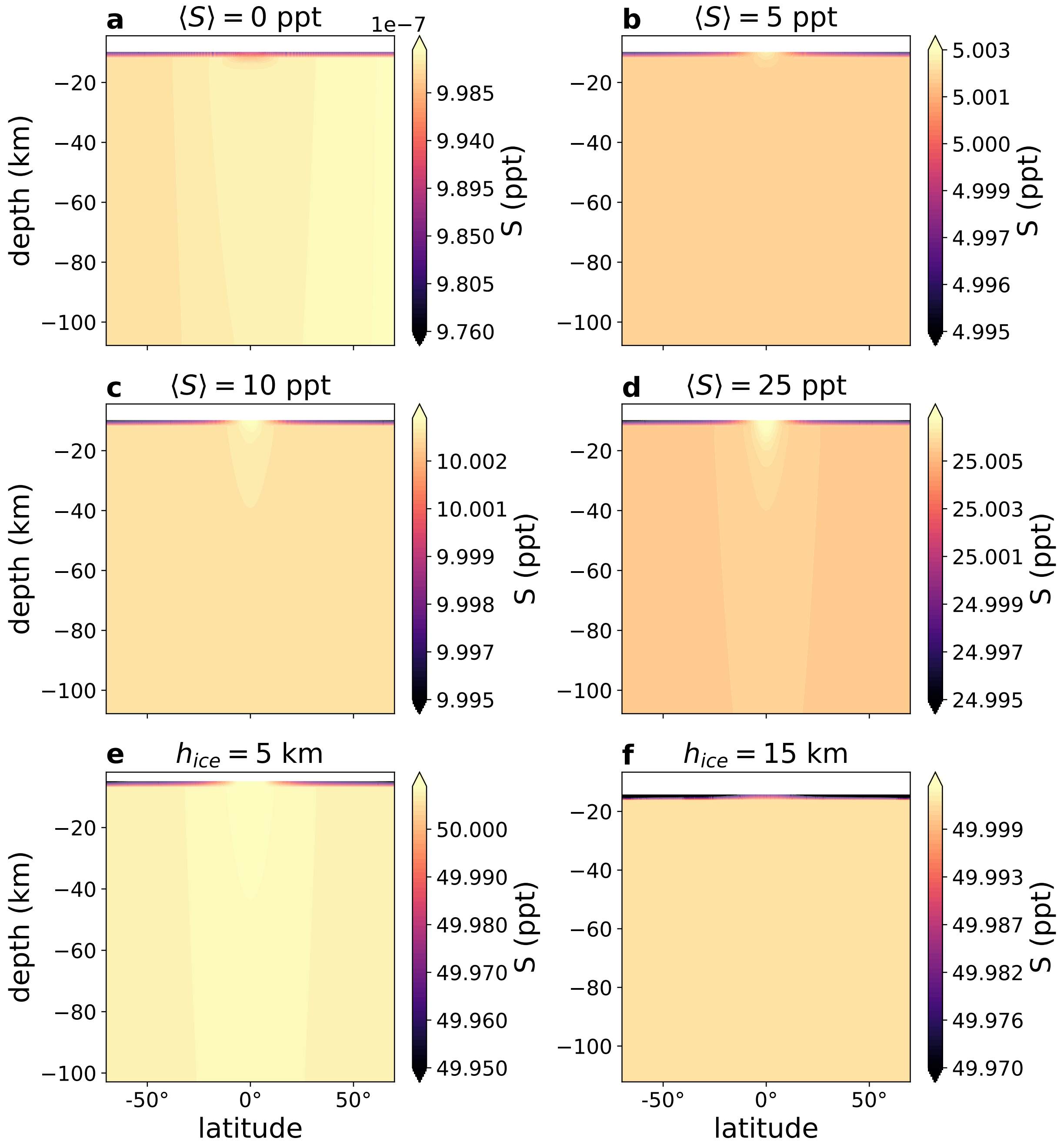

Supplementary Figure 2: **Sensitivity tests–salinity.** Same as Supplementary Fig. 1 for salinity, S.

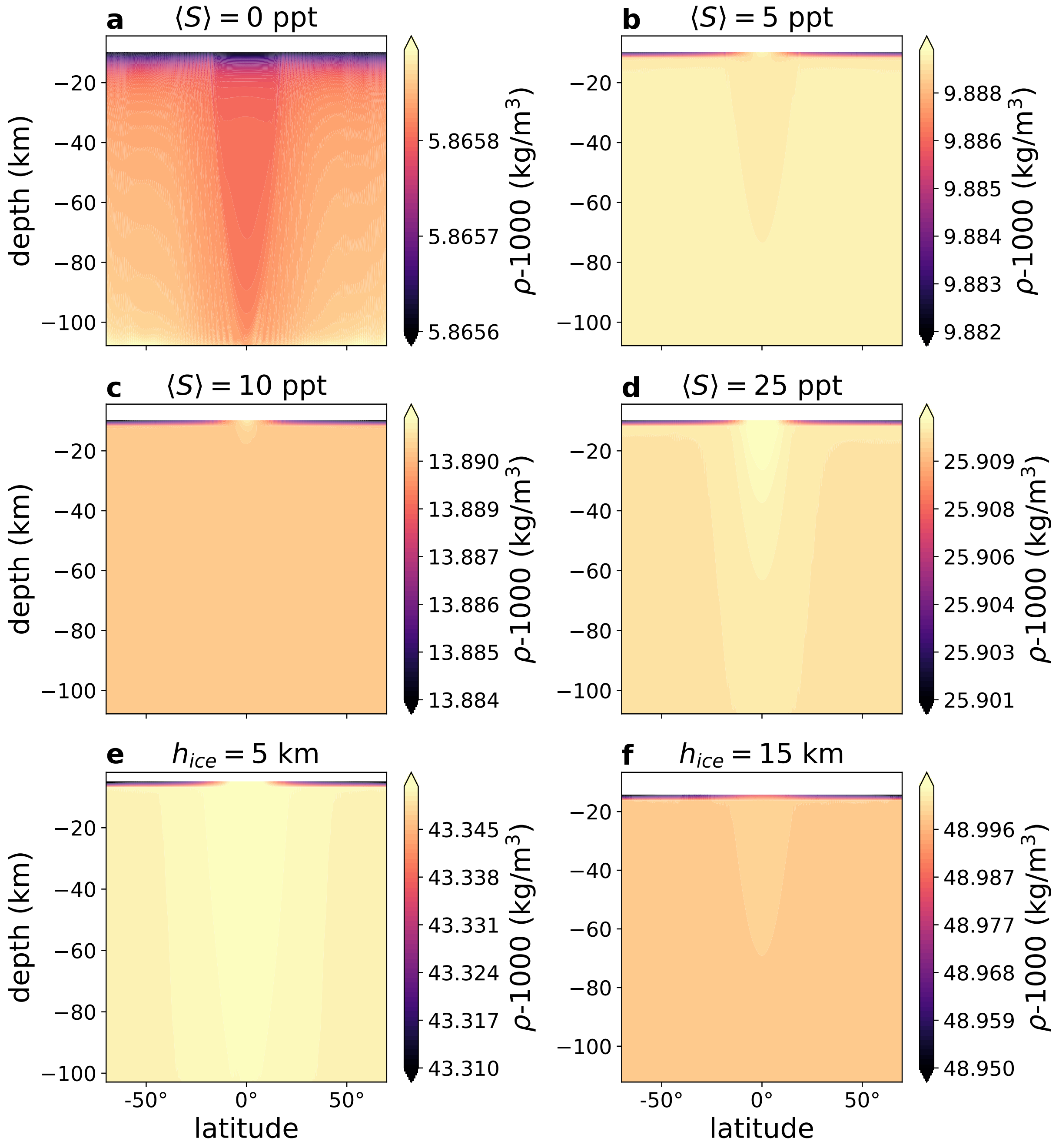

Supplementary Figure 3: **Sensitivity tests–density.** Same as Supplementary Fig. 1 for density, ρ.

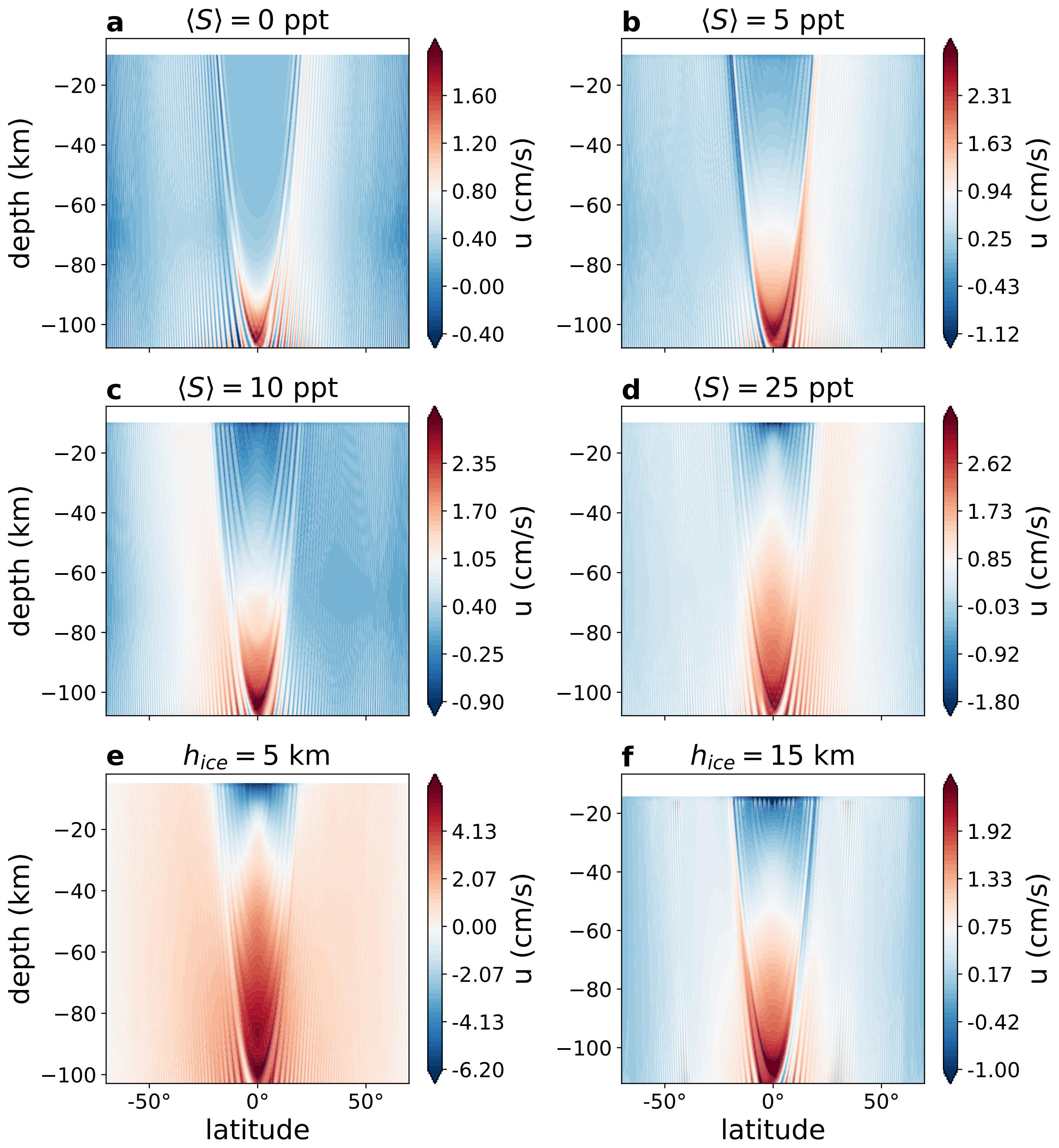

Supplementary Figure 4: **Sensitivity tests–zonal velocity.** Same as Supplementary Fig. 1 for zonal velocity, u.

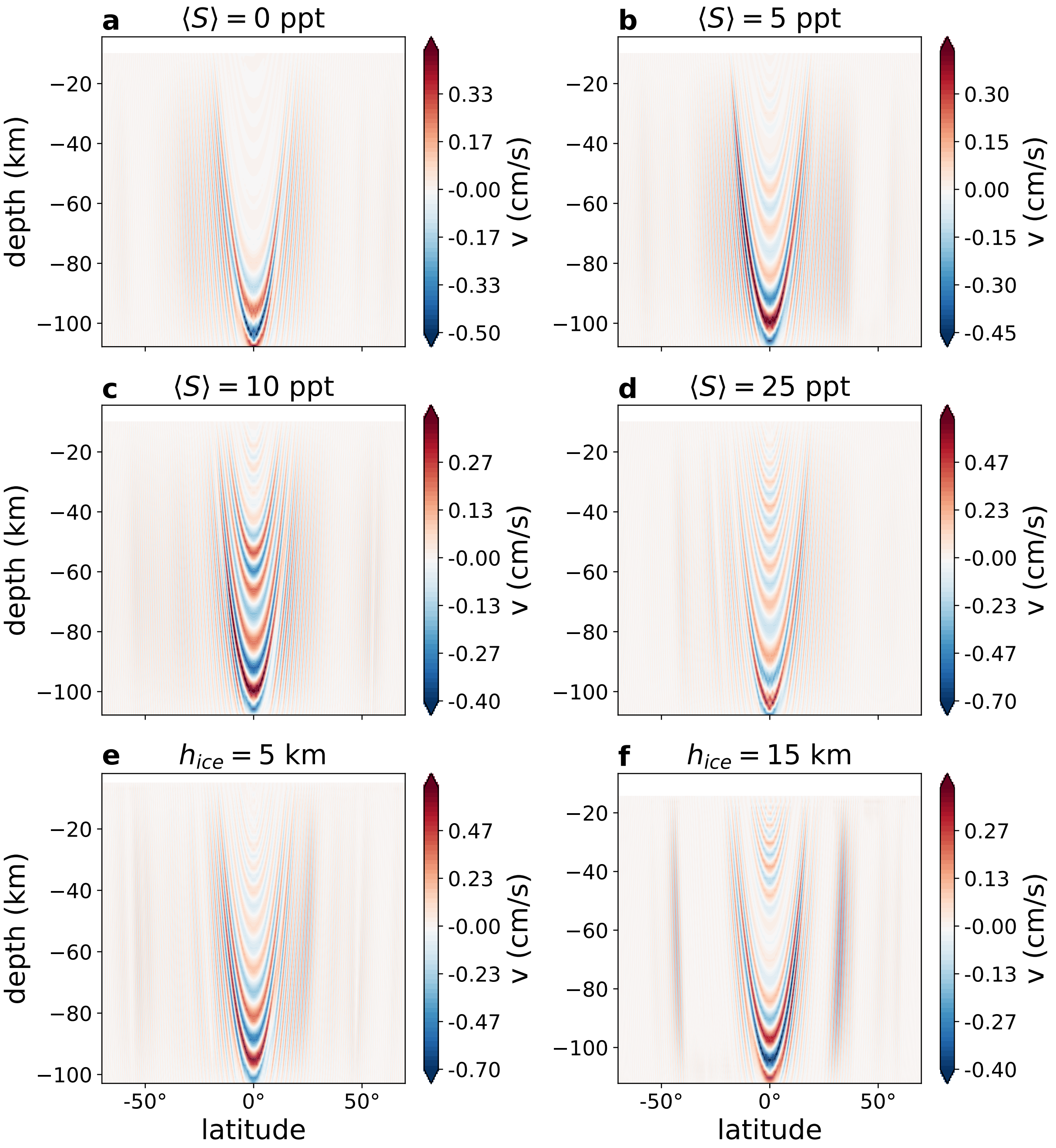

Supplementary Figure 5: **Sensitivity tests–meridional velocity.** Same as Supplementary Fig. 1 for meridional velocity, v.

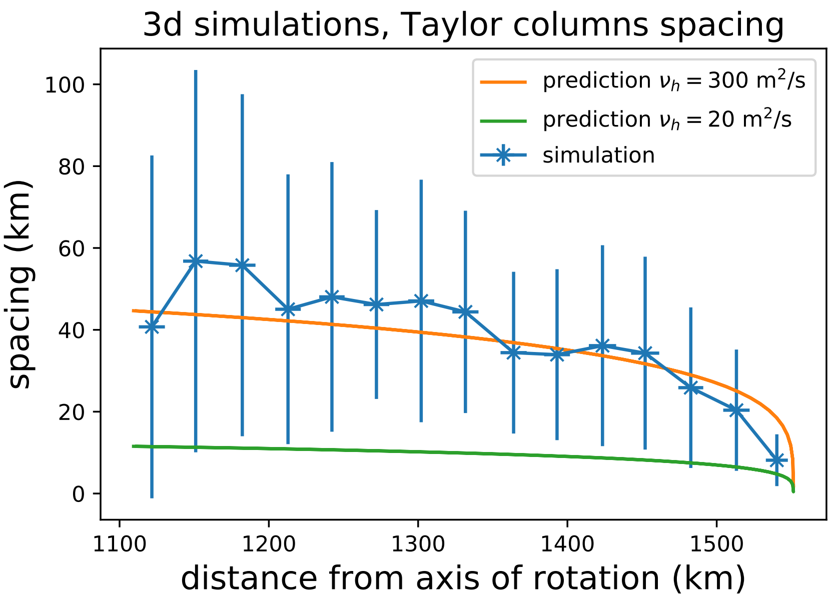

Supplementary Figure 6: **Taylor columns spacing based on the 3d simulation.** The distance between the Taylor columns as a function of the distance from the axis of rotation (in km, blue asterisks). The results are shown for a snapshot at a single time, and the spacing between the columns is calculated for each longitude grid point where then the data was binned using 60 km interval; the std is shown by the vertical bars. The orange line represents the predicted spacing with an eddy viscosity coefficient of $\nu_h = 300\ \mathrm{m^2s^{-1}}$ which fits the numerical values. This eddy coefficient is 15 times larger than the explicit viscosity coefficient used in the numerical simulation (green line), suggesting that the eddy viscosity coefficient due to the explicitly resolved eddy motions in the 3d simulation is 15 times larger than the explicit one.

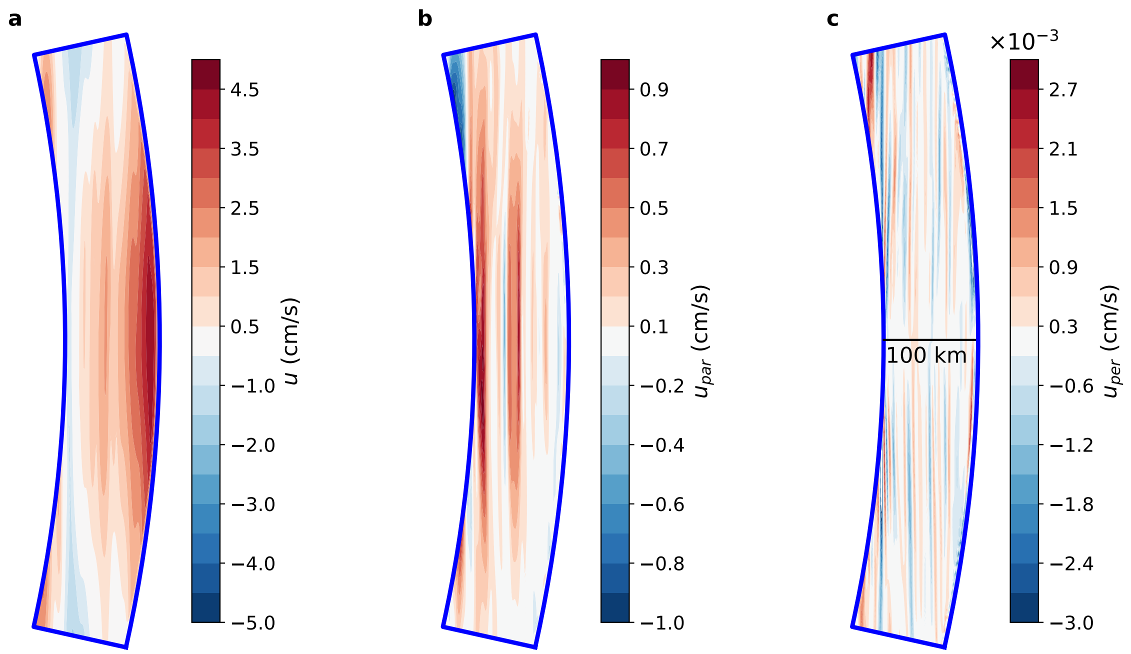

Supplementary Figure 7: **Taylor columns in the 3d simulation.** The velocity components are shown in a spherical projection to supplement the depth-latitude projection in Fig. 5 in the main text: Zonal mean of **a** zonal velocity, u, **b** velocity parallel to the axis of rotation, u_{par}, and **c** velocity perpendicular to the axis of rotation, u_{per}. The latitudinal extent of the plots is from 12.5°S to 12.5°N and the depth extent is 100 km.

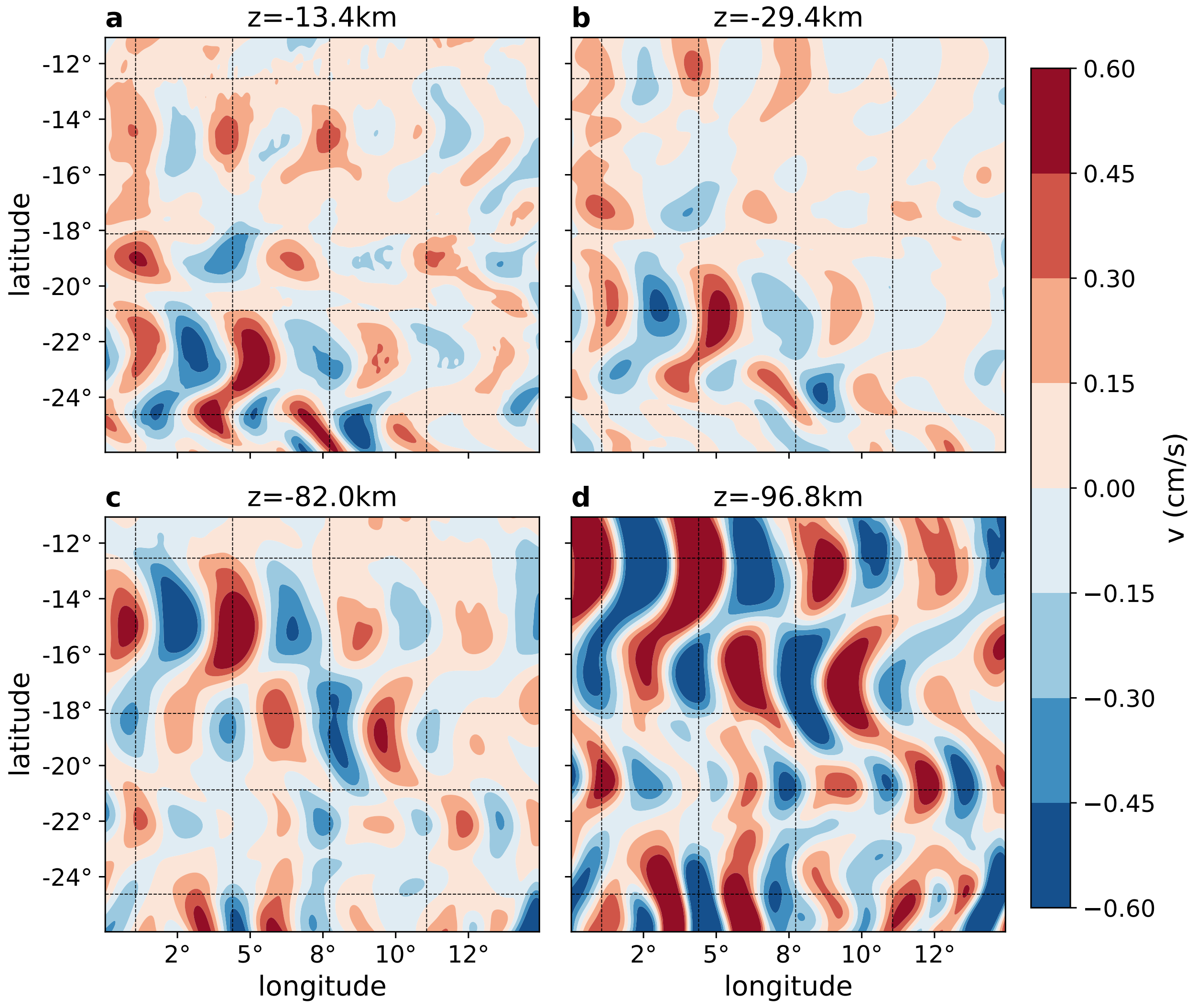

Supplementary Figure 8: **Structure of 3d Taylor columns–longitude-latitude sections.** Longitude-latitude plots of the meridional velocity, v, (in cm s^{-1}) at different depth of **a** $z = -13.4$ km, **b** $z = -29.4$, **c** $z = -82$ km, and **d** $z = -96.8$ km. The vertical dashed lines indicate the zonal sections plotted in Supplementary Fig. 9 while the horizontal dashed lines indicate the zonal sections plotted in Supplementary Fig. 10. The dotted curved line indicates a line parallel to the axis of rotation.

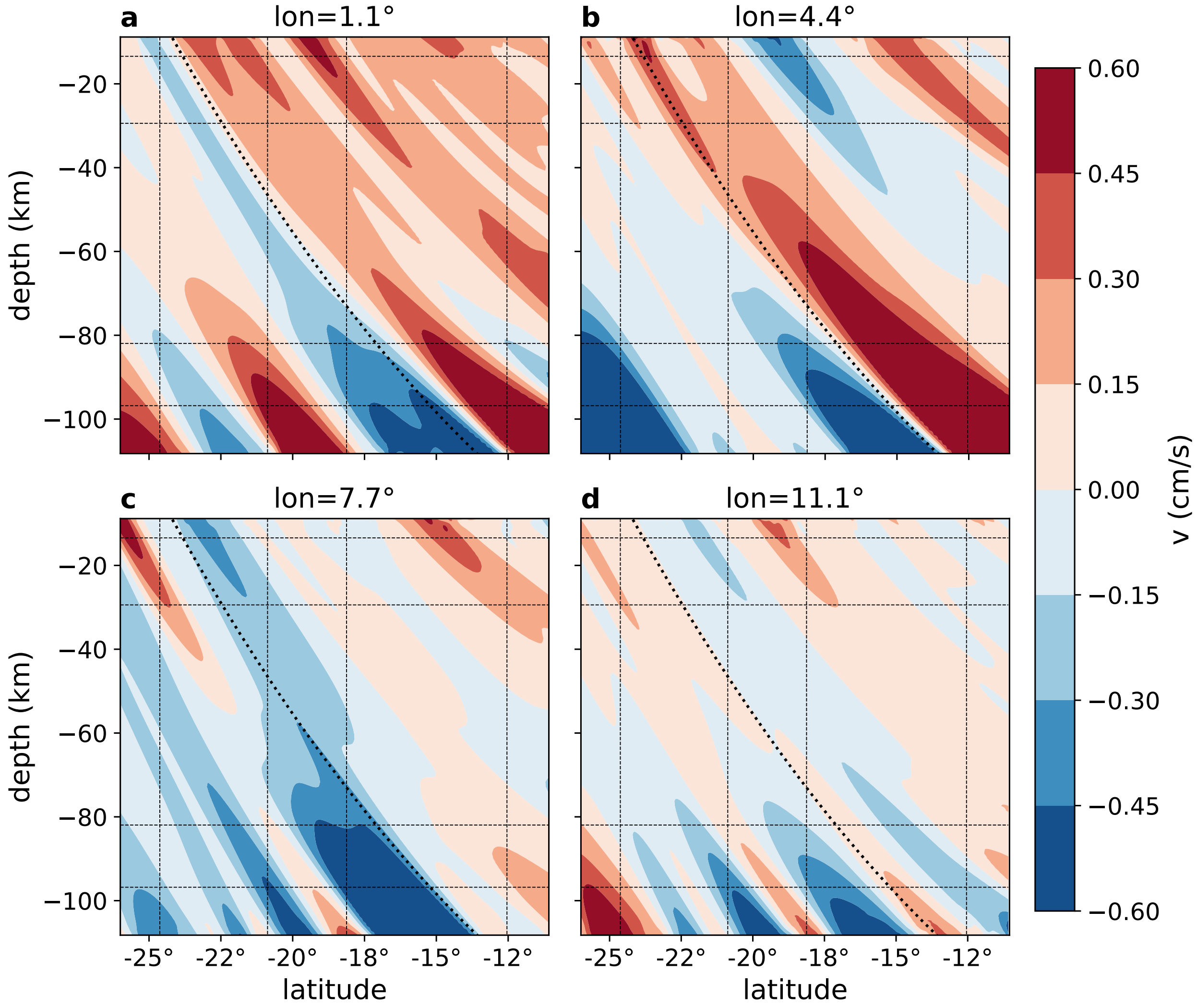

Supplementary Figure 9: **Structure of 3d Taylor columns–latitude-depth sections.** Longitude-depths plots of the meridional velocity, v, (in cm s^{-1}) at different longitudes of **a** 1.1°, **b** 4.4°, **c** 7.7°, and **d** 11.1°. The vertical dashed lines indicate the meridional sections plotted in Supplementary Fig. 10 while the horizontal dashed lines indicate the depth sections plotted in Supplementary Fig. 8. The dotted curved line indicates a line parallel to the axis of rotation.

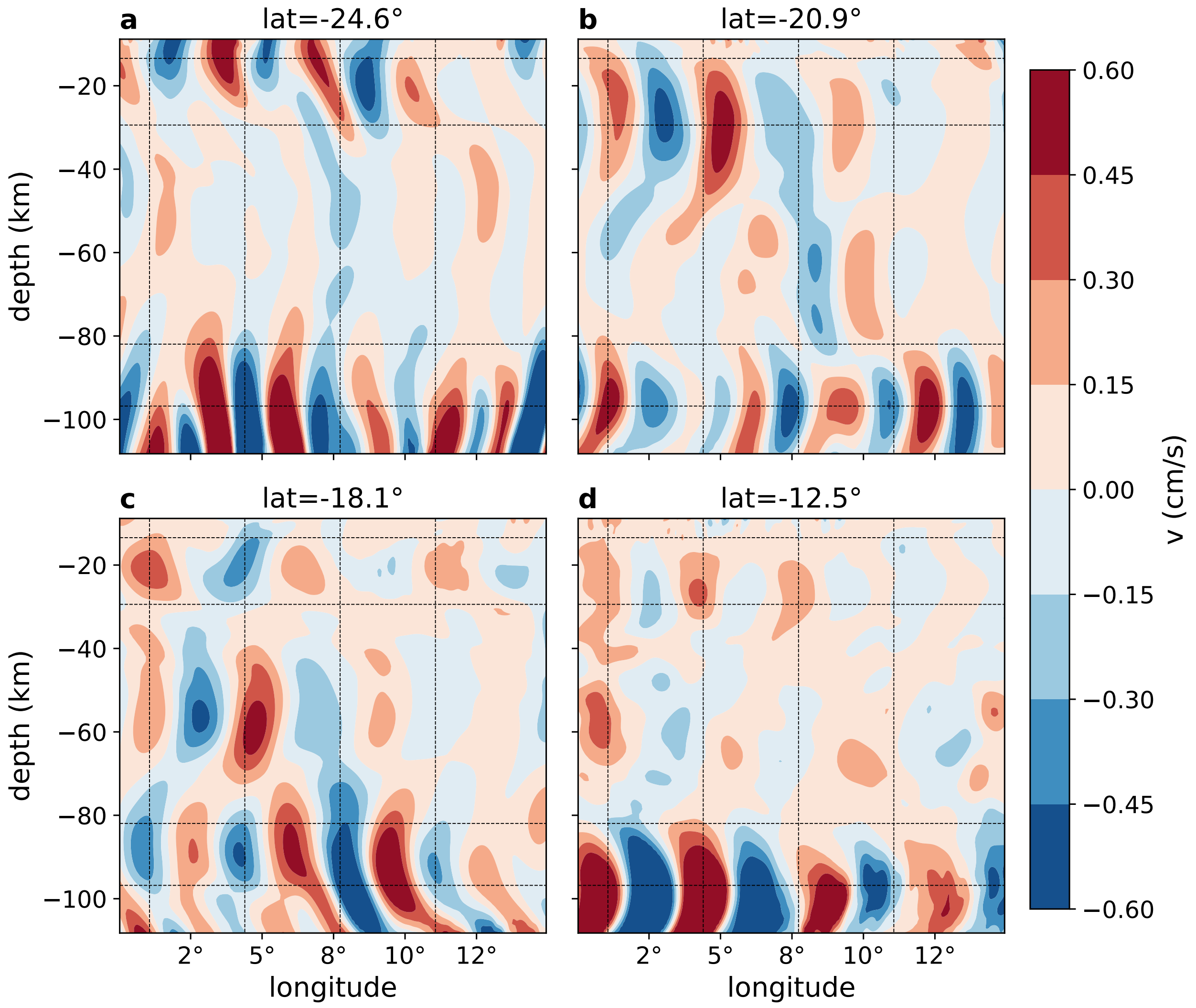

Supplementary Figure 10: **Structure of 3d Taylor columns–longitude-depth sections.** Longitude-depth plots of the meridional velocity, v, (in cm s^{-1}) at different latitudes of **a** -24.6°, **b** -20.9°, **c** -18.1°, and **d** -12.5°. The vertical dashed lines indicate the zonal sections plotted in Supplementary Fig. 9 while the horizontal dashed lines indicate the depth sections plotted in Supplementary Fig. 8. The dotted curved line indicates a line parallel to the axis of rotation.

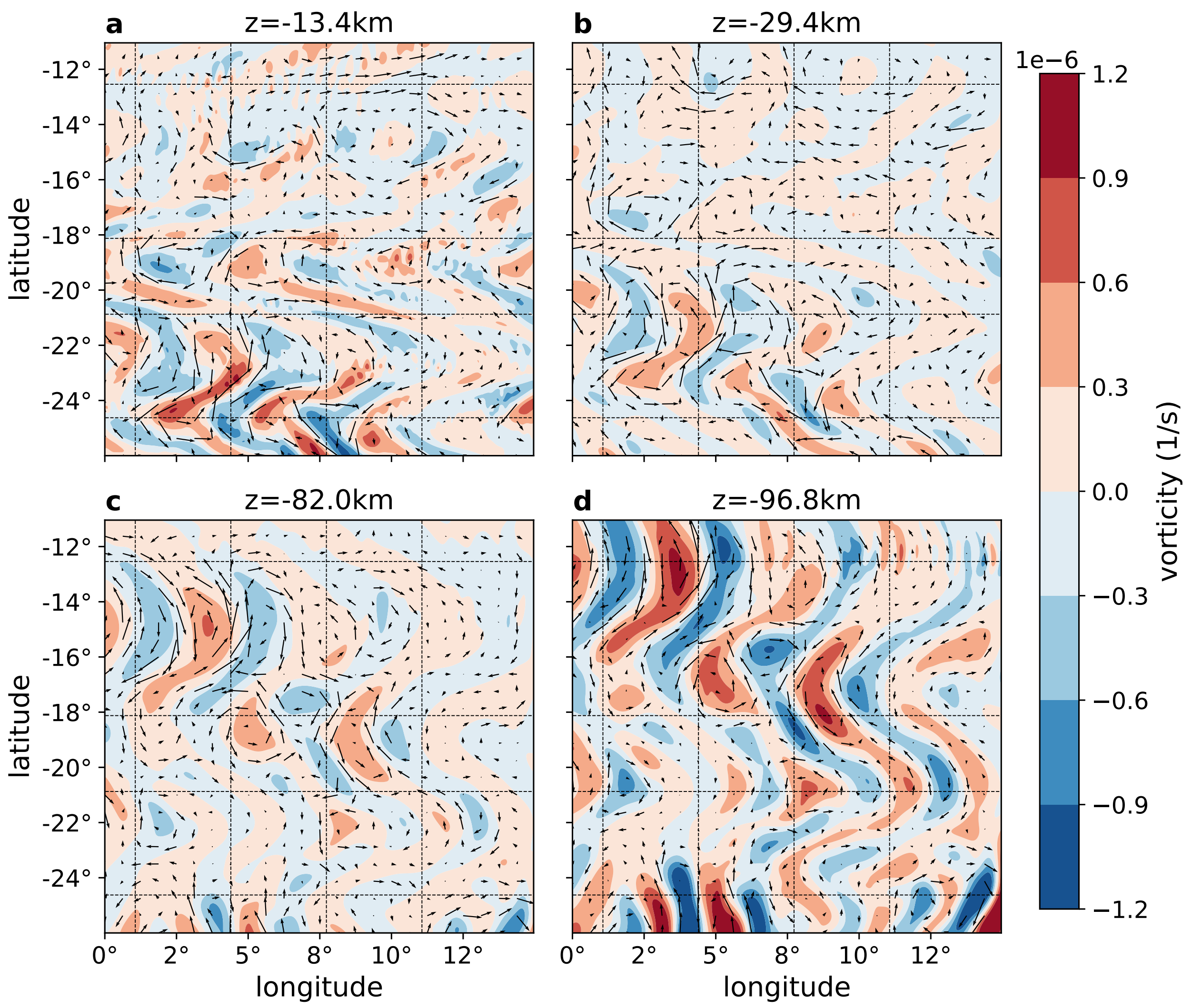

Supplementary Figure 11: **Structure of 3d Taylor columns–longitude-latitude section of vorticity.** Same as Supplementary Fig. 8 for vorticity (in s^{-1}).

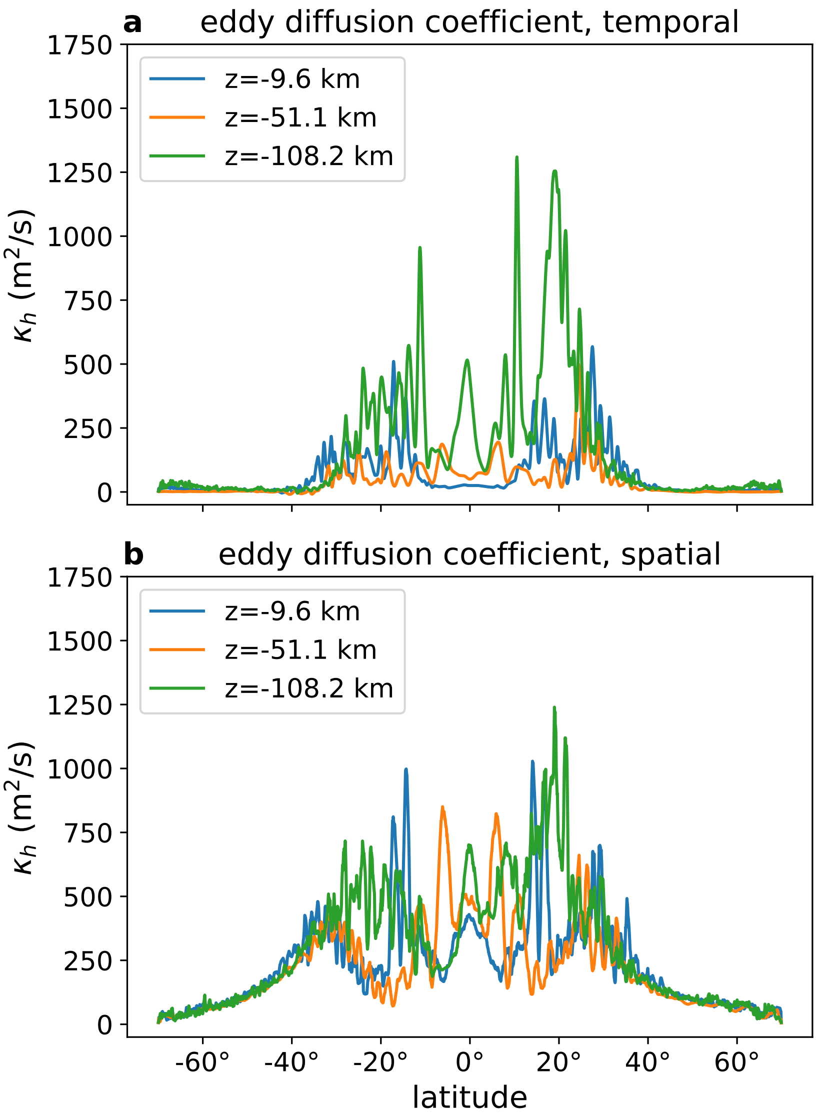

Supplementary Figure 12: **Estimating the eddy coefficients.** The estimate is based on the 3d simulation using the **a** temporal auto-correlation function and **b** spatial auto-correlation function. The zonal mean at the top ($z = -9.6$ km, blue), middle ($z = -51.1$ km, orange), and bottom ($z = -108.2$ km, green) of the ocean is plotted versus latitude where the estimated diffusion coefficient, κ_h, is significantly smaller at the high latitudes. The estimated eddy viscosity coefficient is equal to or larger than the eddy diffusion coefficient.

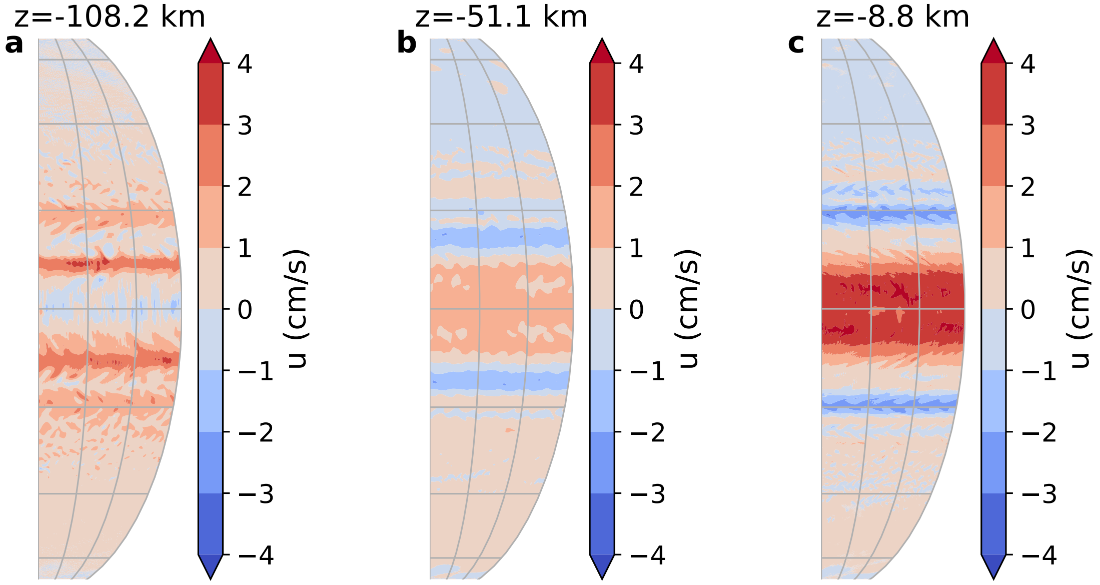

Supplementary Figure 13: **Zonal velocity, u, in 3d simulation.** Shown in a spherical (longitude-latitude) projection at the **a** bottom ($z = -108.2$ km), **b** middle ($z = -51.1$ km), and **c** top ($z = -8.8$ km) of the ocean. The grid line spacing is 10° in the zonal direction and 20° in the meridional direction. The figure depicts a "Jupiter-like" structure of alternating zonal jets as was previously predicted[1].

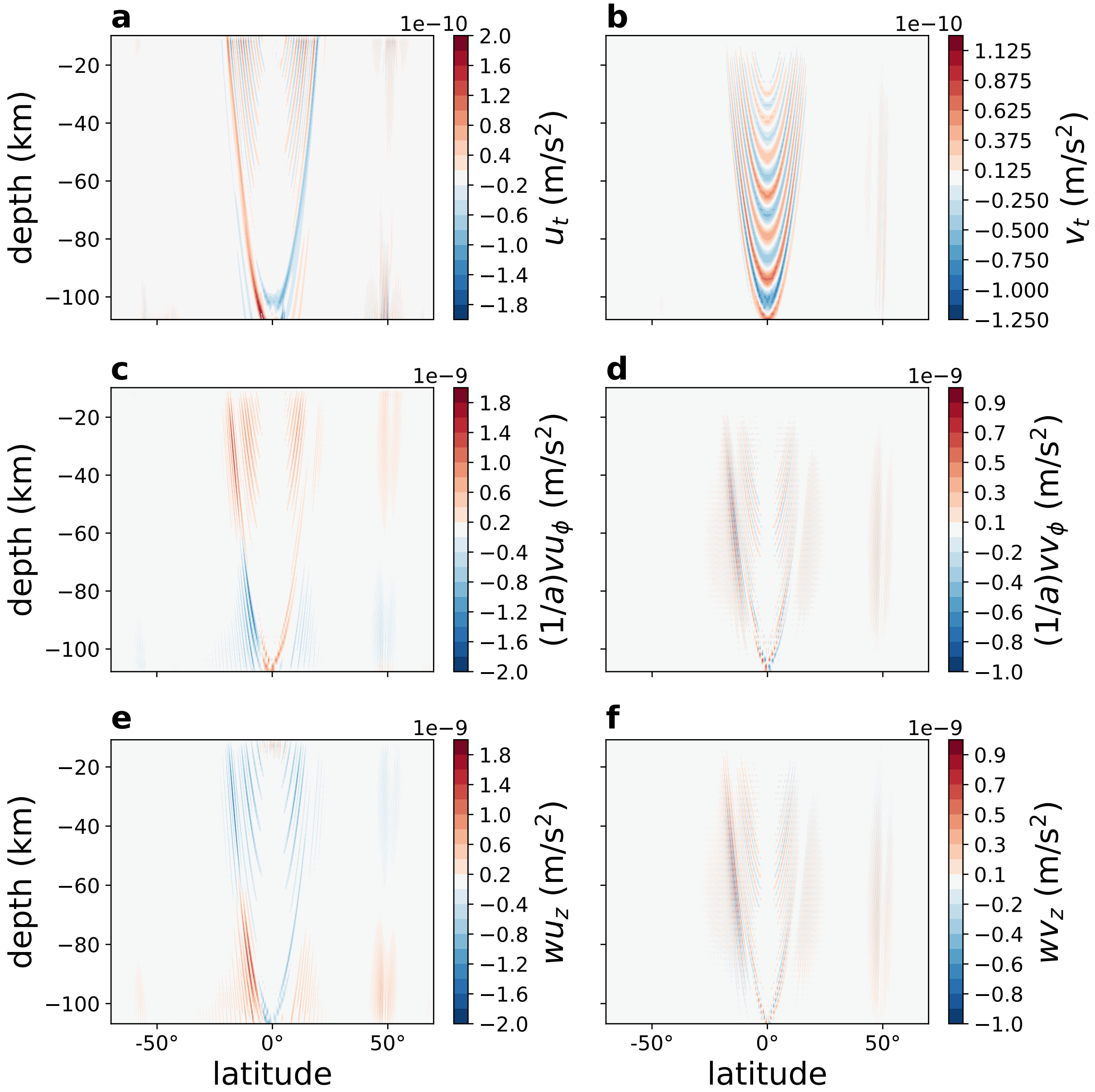

Supplementary Figure 14: **Time and advection terms in the momentum equations.** Snapshots of the: **a** time derivative of the zonal velocity, u_t, **b** time derivative of the zonal velocity, v_t, **c** meridional advection of the zonal velocity, $\frac{1}{a}vu_\phi$, **d** meridional advection of the meridional velocity, $\frac{1}{a}vv_\phi$, **e** vertical advection of the zonal velocity, $\frac{1}{a}wu_z$, and **f** vertical advection of the moridional velocity, $\frac{1}{a}wv_z$. ϕ, z, t are the meridional, vertical, and time coordinates, u, v, w are the zonal, meridional, and meridional velocities, and a is the radius of Europa.

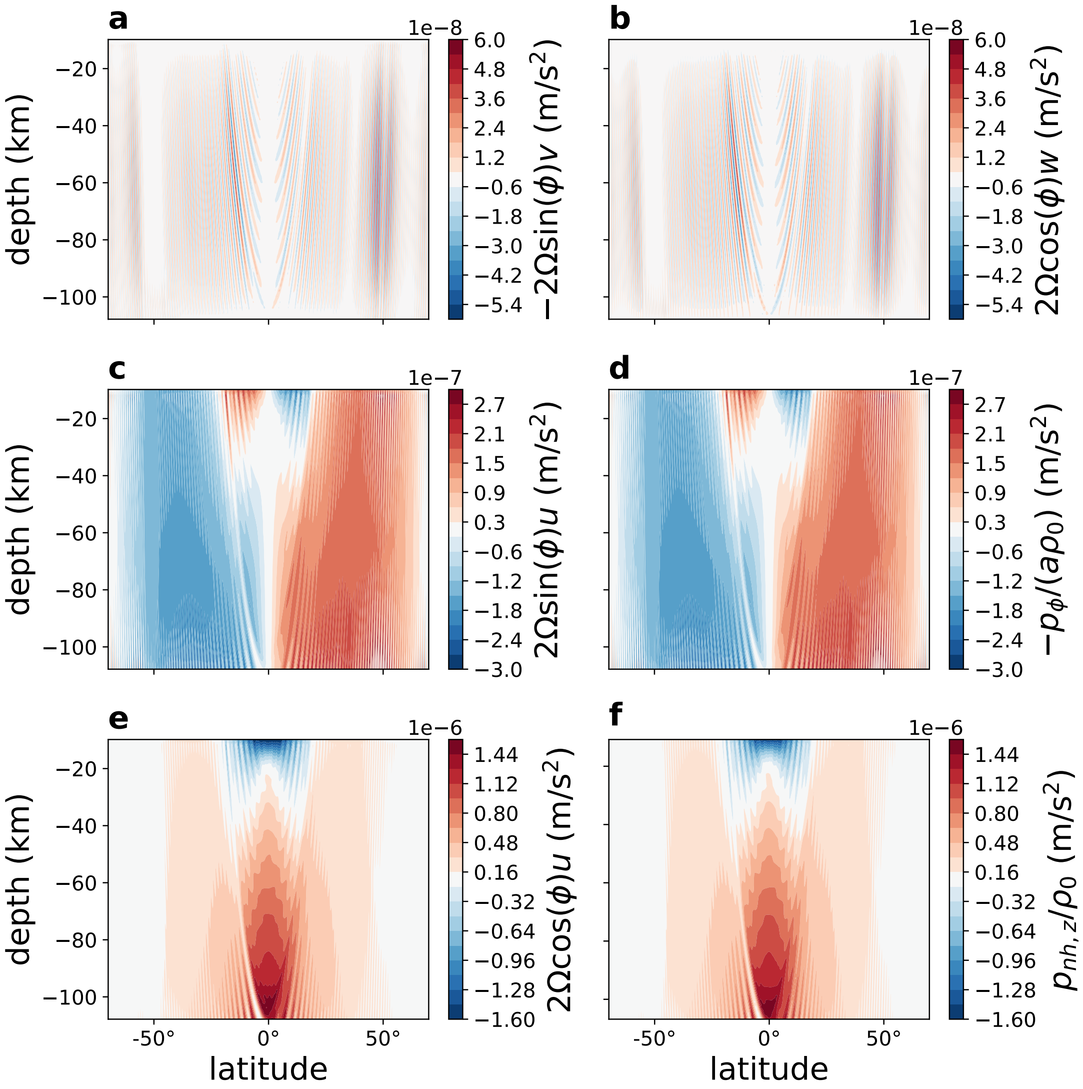

Supplementary Figure 15: **Coriolis and pressure terms in the momentum equations.** Snapshots of the: **a** Coriolis term, $-2\Omega\sin(\phi)v$, **b** co-Coriolis term, $2\Omega\cos(\phi)w$, **c** Coriolis term, $2\Omega\sin(\phi)u$, **d** meridional pressure gradient, $-\frac{1}{a\rho_0}p_\phi$, **e** co-Coriolis term, $2\Omega\cos(\phi)u$, and **f** vertical (non-hydrostatic) pressure gradient term, $\frac{1}{\rho_0}p_{nh,z}$. Panels **e** and **f** depict the most dominant terms in the vertical momentum equation. ϕ, z are the meridional and vertical coordinates, u, v, w are the zonal, meridional, and meridional velocities, p, p_{nh} are the total and non-hydrostatic pressures, a is the radius of Europa, Ω is the rotation rate of Europa, and ρ_0 is the reference density of Europa's ocean.

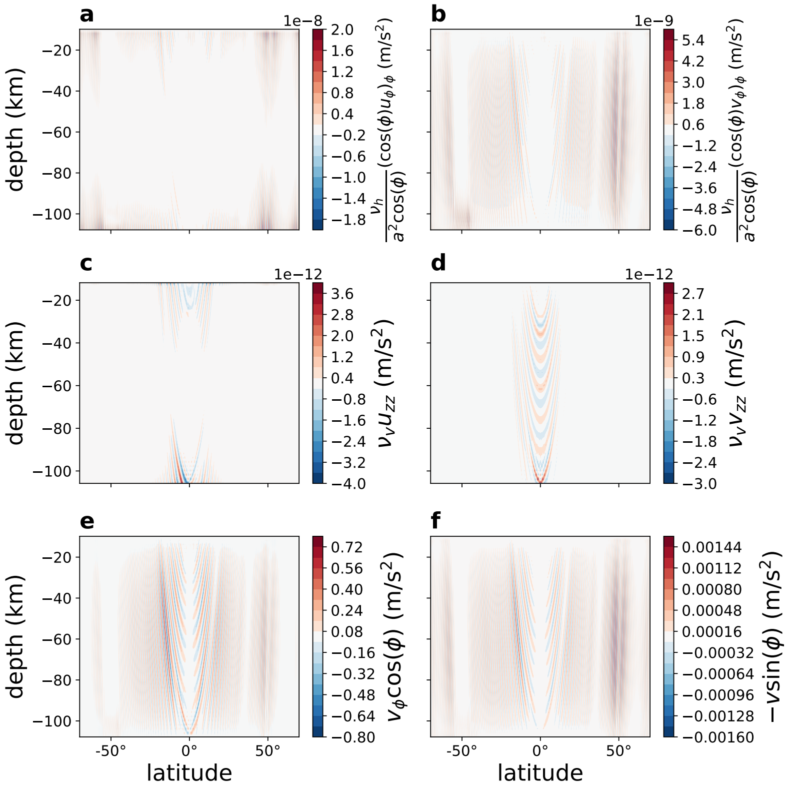

Supplementary Figure 16: **Viscosity terms in the momentum equations and terms in the continuity equation.** Snapshots of the: **a** meridional viscosity of the zonal velocity, $\frac{\nu_h}{a^2\cos(\phi)}(\cos(\phi)u_\phi)_\phi$, **b** meridional viscosity of the meridional velocity, $\frac{\nu_h}{a^2\cos(\phi)}(\cos(\phi)v_\phi)_\phi$, **c** vertical viscosity of the zonal velocity, $\nu_v u_{zz}$, **d** vertical viscosity of the meridional velocity, $\nu_v v_{zz}$. Panels **e** and **f** show terms in the continuity equation and justify our approximation $\frac{1}{a\cos(\phi)}(v\cos(\phi))_\phi + w_z = 0$, that $(v\cos(\phi))_\phi = v_\phi\cos(\phi) - v\sin(\phi) \approx v_\phi\cos(\phi)$ since $v_\phi\cos(\phi) \gg v\sin(\phi)$. ϕ, z are the meridional and vertical coordinates, u, v, w are the zonal, meridional, and vertical velocities, ν_h, ν_v are the horizontal and vertical viscosity coefficients, and a is the radius of Europa. Based on Supplement Figs. 14–16, the most dominant terms in the zonal momentum equation are the Coriolis terms (Supplementary Fig. 15a,b), the most dominant terms in the meridional momentum equation are the Coriolis and the pressure gradient terms (Supplementary Fig. 15c,d) which nearly balance each other. The next dominant terms in the zonal and meridional momentum equations are the horizontal viscosity terms (Supplementary Fig. 16a,b).

Supplementary References

1. Vance, S. & Goodman, J. Oceanography of an ice-covered moon. In Pappalardo, R. T., McKinnon, W. B. & Khurana, K. (eds.) *Europa*, 459–482 (The University of Arizona Press, Tucson, AZ, 2009).
