# Supplementary material for: Dynamic Europa ocean shows transient Taylor columns and convection driven by ice melting and salinity
Source: Nat Commun. 2021 Nov 4;12:6376. doi: 10.1038/s41467-021-26710-0 (PMC8569204; doi:10.1038/s41467-021-26710-0)
Supplement: Supplementary file 2 — Description of Additional Supplementary Files [file 41467_2021_26710_MOESM2_ESM.docx]

Description of Additional Supplementary Files

File Name: Supplementary Video 1

Description: Animation showing top and bottom convection processes. This animation is based on the 3d temperature field.

File Name: Supplementary Video 2

Description: Animation showing eddies and turbulence at the top of the ocean. This animation is based on the 3d temperature field.
