# Supplementary figures and images for: Dynamic Europa ocean shows transient Taylor columns and convection driven by ice melting and salinity
Source: Nat Commun. 2021 Nov 4;12:6376. doi: 10.1038/s41467-021-26710-0 (PMC8569204; doi:10.1038/s41467-021-26710-0)

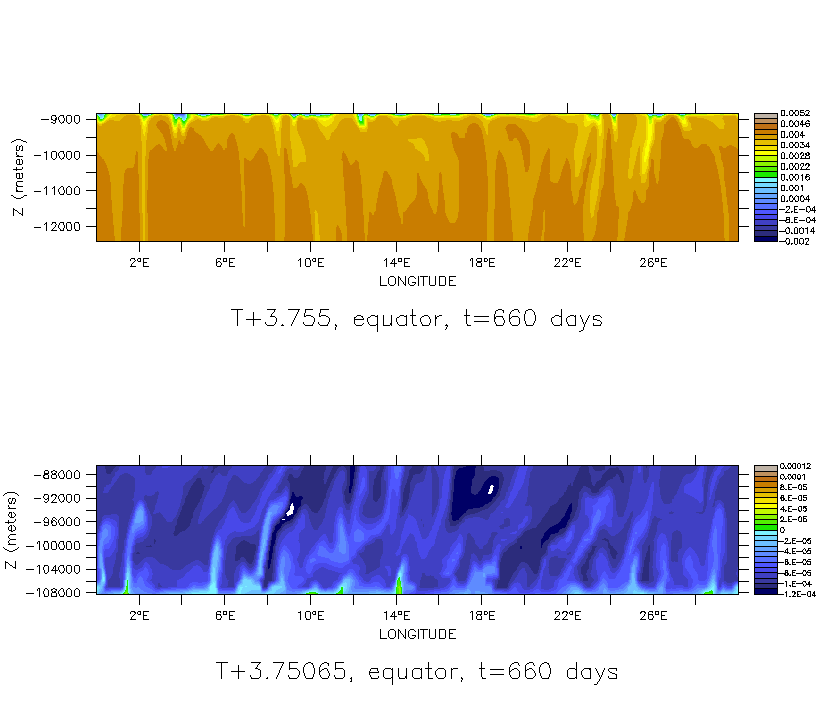

Supplement: Supplementary file 3 — Supplementary Video 1 [file 41467_2021_26710_MOESM3_ESM.gif]

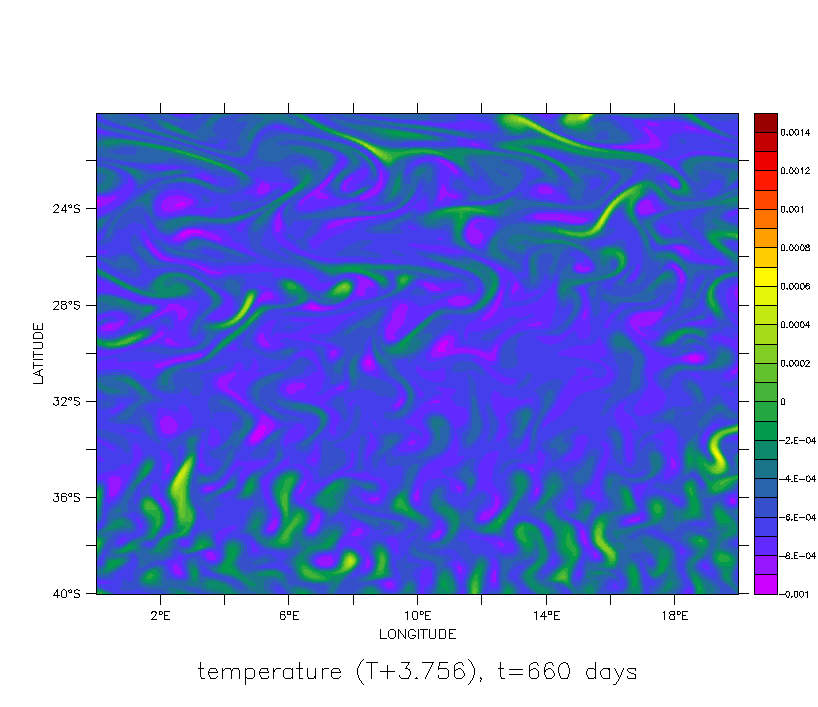

Supplement: Supplementary file 4 — Supplementary Video 2 [file 41467_2021_26710_MOESM4_ESM.gif]
